# Proteomic Response of *Hordeum vulgare* cv. Tadmor and *Hordeum marinum* to Salinity Stress: Similarities and Differences between a Glycophyte and a Halophyte

**DOI:** 10.3389/fpls.2016.01154

**Published:** 2016-08-03

**Authors:** Lucie Maršálová, Pavel Vítámvás, Radovan Hynek, Ilja T. Prášil, Klára Kosová

**Affiliations:** ^1^Department of Biochemistry and Microbiology, Faculty of Food and Biochemical Technology, University of Chemistry and TechnologyPrague, Czech Republic; ^2^Laboratory of Plant Stress Biology and Biotechnology, Division of Crop Genetics and Breeding, Crop Research InstitutePrague, Czech Republic

**Keywords:** glycophyte, halophyte, salinity, proteome, stress acclimation, stress damage, *Hordeum marinum*, *Hordeum vulgare*

## Abstract

Response to a high salinity treatment of 300 mM NaCl was studied in a cultivated barley *Hordeum vulgare* Syrian cultivar Tadmor and in a halophytic wild barley *H. marinum*. Differential salinity tolerance of *H. marinum* and *H. vulgare* is underlied by qualitative and quantitative differences in proteins involved in a variety of biological processes. The major aim was to identify proteins underlying differential salinity tolerance between the two barley species. Analyses of plant water content, osmotic potential and accumulation of proline and dehydrin proteins under high salinity revealed a relatively higher water saturation deficit in *H. marinum* than in *H. vulgare* while *H. vulgare* had lower osmotic potential corresponding with high levels of proline and dehydrins. Analysis of proteins soluble upon boiling isolated from control and salt-treated crown tissues revealed similarities as well as differences between *H. marinum* and *H. vulgare*. The similar salinity responses of both barley species lie in enhanced levels of stress-protective proteins such as defense-related proteins from late-embryogenesis abundant family, several chaperones from heat shock protein family, and others such as GrpE. However, there have also been found significant differences between *H. marinum* and *H. vulgare* salinity response indicating an active stress acclimation in *H. marinum* while stress damage in *H. vulgare*. An active acclimation to high salinity in *H. marinum* is underlined by enhanced levels of several stress-responsive transcription factors from basic leucine zipper and nascent polypeptide-associated complex families. In salt-treated *H. marinum*, enhanced levels of proteins involved in energy metabolism such as glycolysis, ATP metabolism, and photosynthesis-related proteins indicate an active acclimation to enhanced energy requirements during an establishment of novel plant homeostasis. In contrast, changes at proteome level in salt-treated *H. vulgare* indicate plant tissue damage as revealed by enhanced levels of proteins involved in proteasome-dependent protein degradation and proteins related to apoptosis. The results of proteomic analysis clearly indicate differential responses to high salinity and provide more profound insight into biological mechanisms underlying salinity response between two barley species with contrasting salinity tolerance.

## Introduction

Salinity represents an important threat to agricultural production worldwide, especially in hot arid and semi-arid areas. It is estimated that salinity affects ca 7% of agricultural land globally while in irrigated agriculture, it is nearly one third of irrigated aricultural land and the salt-affected area is steadily increasing. Plants exposed to increased salt levels generally reveal a two-phase salinity response: first, a non-specific osmotic effect, i.e., cellular dehydration caused by a decreased water potential of salt solution; and second, a salt-specific ionic effect, i.e., an enhanced salt ion influx into cell cytoplasm leading to significant alterations in cell ion homeostasis (Zhu, 2001; Munns, 2002). Osmotic effect is common to all dehydration stresses and leads to an accumulation of osmolytes in cell cytoplasm to counteract the adverse effects on plant water uptake. In contrast, ionic effect is specific for salinity stresses and lies in enhanced concentrations of salt ions, namely Na^+^, in soil water solution which results in enhanced salt ion levels in cell cytoplasm resulting in an activation of salt ion exclusion and compartmentation mechanisms (Munns, 2002; Munns and Tester, 2008).

Barley (*H. vulgare*) is a relatively dehydration- and salt-tolerant cereal, the most tolerant of cultivated Triticeae, which can grow up to 250 mM NaCl (Colmer et al., 2006). However, several wild Triticeae species are halophytes, i.e., they can grow in areas with salt concentrations approaching those in sea water, i.e., around 500 mM NaCl, and can complete their life cycle at salinities above 200 mM NaCl (Flowers, 2004; Colmer et al., 2006). In Triticeae, crown tissues are crucial for the whole plant survival. Sea barleygrass (*H. marinum*) is a wild halophytic Triticeae species growing in salt marshes and other coastal areas in Mediterranean region and the Middle East. Previous experiments aimed at an investigation of *H. marinum* salt response mechanisms revealed that *H. marinum* maintained low levels of salt ions in the shoot indicating efficient ion exclusion mechanisms at the level of xylem transport (Garthwaite et al., 2005; Islam et al., 2007). In contrast, cultivated barley *H. vulgare* reveals relatively high Na^+^ levels in shoots when exposed to salinity resulting in enhanced Na^+^ vacuolar compartmentation. These processes seem to be compensated by cytosolic osmolyte accumulation leading to decreased shoot osmotic potential levels (Garthwaite et al., 2005).

Proteins play a crucial role in plant stress response since they are directly involved in the processes aimed at an enhancement of stress tolerance. Similarly to other stresses, salinity induces complex adaptations including an enhanced abundance of several stress-related proteins (dehydration-induced proteins, ion transporters, and ROS scavenging enzymes) as well as changes in cell signaling, gene expression, cellular metabolism, and regulatory processes. Despite an increasing number of proteomic studies aimed at crop stress response (reviewed in Kosová et al., 2011, 2013a,b, 2014a; Komatsu et al., 2014), there have been published only a few studies aimed at proteome response to salinity in halophytes (reviewed in Kumari et al., 2015) as well as a comparison of proteome response between related plant species with contrasting salt tolerance (a glycophyte vs a halophyte; reviewed in Kosová et al., 2013d). The few studies aimed at comparison of salt response between a glycophyte and a halophyte at transcript and protein levels include a comparison of *Arabidopsis thaliana* and *Thellungiella halophila* (Taji et al., 2004; Gong et al., 2005; Pang et al., 2010), common wheat *Triticum aestivum* and *T. aestivum-Thinopyrum ponticum* amphiploid (Wang et al., 2008; Peng et al., 2009), and rice and its wild relative *Porteresia coarctata* (Sengupta and Majumder, 2009). The results of the few studies indicate that salt-tolerant species revealed higher constitutive levels of stress-related proteins (ROS scavenging enzymes, salt ion transporters SOS1, V-ATPase), and, under stress, thus they revealed relatively higher levels of anabolism-related proteins (RubisCO activase and other proteins involved in photosynthesis such as OEE proteins) than salt-sensitive species. In contrast, salt-sensitive species reveal relatively higher levels of catabolism-related proteins such as glycolytic and respiratory enzymes. However, most important aspects of the differential salinity response between glycophytes and halophytes still remain unexplored.

The manuscript represents the first study aimed at a comparison of proteome response to high salinity (300 mM NaCl) in cultivated barley *H. vulgare* and its halophytic relative *H. marinum*. High salinity (300 mM NaCl) was applied in order to induce stress response in both *H. vulgare* and *H. marinum*. First, both salt acclimation treatments as well as one-step direct transfers from control to high salinity conditions were applied in order to investigate plant stress response by using simple physiological assessments of tissue dehydration (WSD; PWC; leaf sap osmotic potential ψ_s_), osmolyte accumulation (proline, dehydrins) and impairment of photosynthetic apparatus (maximum quantum yield efficiency *F*_v_/*F*_m_). Next, an one-step transfer from control to high salinity (0–300 mM NaCl) was chosen for proteomic analysis since only this kind of salinity treatment induced a significant stress response in salt-tolerant *H. marinum*. An analysis of boiling-stable proteins enriched fractions led to an identification of several stress-responsive proteins underlying differential salinity tolerance in salt-sensitive *H. vulgare* and salt-tolerant *H. marinum*, respectively, which would help us to understand differential mechanisms employed in salinity response by related species of glycophytes and halophytes.

## Materials and Methods

### Plant Material and Growth Conditions

*Hordeum vulgare* cv. Tadmor is a black-seeded barley cultivar derived from a Syrian landrace Arabi Aswad by ICARDA, Aleppo, Syria. *H. marinum* ssp. *gussoneanum* is a wild halophytic barley species adapted to salt marshes and other habitats with enhanced soil salinity. Seeds of *H. vulgare* cv. Tadmor were obtained from Dr. L. Holková, Mendel University, Brno, Czech Republic. Seeds of *H. marinum* ssp. *gussoneanum*, accession H818 from Iran, were obtained from Nordic Gene Bank, Alnarp, Sweden. Seeds of both barley species were surface sterilized with 1% (m/v) sodium hypochlorite for 5 min followed by rinsing with distilled water. The seeds were let germinate on moist filter paper in the dark for 5 days under temperature of 20°C and then plants were grown hydroponically in continuously aerated pots in a growth chamber (*Tyler T-16/4*, Budapest, Hungary) under an irradiance of 350 μmol m^-2^ s^-1^, a 12-h photoperiod, and temperature of 20°C. The pots were filled with a commercially available solution *Hydropon* (*Lovochemie*, Lovosice, Czech Republic) corresponding to the Hoagland 3 nutrient solution including microelements, the final dilution was 1:200 (v/v); pH of the solution was adjusted to 6.5 by addition of KOH as described previously (Prášil et al., 2005). The hydroponical solution was changed every 3rd day in order to prevent nutrient depletion. All plants were first exposed to the control treatment of 0.2 mM NaCl (osmotic potential of the control solution ψ = -0.095 MPa) for 7 days similarly to previous publications (Islam et al., 2007; Kosová et al., 2015) due to a halophytic nature of *H. marinum*. NaCl was added to the hydroponical solution every day in a gradually increasing manner by 50 mM NaCl up to the final concentrations of 100 mM NaCl (100; a moderate salt stress ψ = -0.608 MPa) and 300 mM NaCl (300; a high salt stress ψ = -1.64 MPa), respectively. The alternative way represented direct transfers from 0.2 to 300 mM NaCl (0.2–300) and from 100 to 300 mM NaCl (100–300). All variants were sampled after 7 days of reaching 300 mM NaCl and the transfers, i.e., after 20 days of hydroponic cultivation. A scheme of the whole experiment is given in Supplementary Figure S1.

### Determination of Physiological Parameters and Dehydrin Relative Accumulation

Determination of WSD, osmotic potential (ψ_s_), proline accumulation, chlorophyll fluorescence parameter maximum quantum yield of photosystem II photochemistry (*F*_v_/*F*_m_) and dehydrin relative accumulation was performed as described previously in Kosová et al. (2015). Youngest fully developed leaves were used for the analyses. Briefly, WSD was determined according to Slavík (1963) as WSD (%) = (*W*_T_–*W*_F_)/(*W*_T_–*W*_D_)^∗^100 where *W*_D_ was dry weight, *W*_F_ was fresh weight and *W*_T_ was turgescent weight of the leaf sample. PWC was determined as ml (g) of water per 1 g of dry mass and it was calculated as follows: PWC = (*W*_F_–*W*_D_)/(*W*_D_).

Leaf osmotic potential (ψ_s_) was determined as osmolarity using VAPRO Dew Point Osmometer (WESCOR Inc., MtLogan, UT, USA). Osmotic potential values were calculated using van’t Hoff equation ψ_s_ = -*n*_i_^∗^*c*_i_^∗^R^∗^T where *n*_i_ is the molar amount of the dissociated ions, *c*_i_ is a concentration of a given solute, R is an universal gas constant and T is a thermodynamic temperature (K).

Proline accumulation was determined spectrophotometrically at 520 nm as proline-ninhydrin complex extracted from leaf tissue using a mixture of 2.08 M orthophosphoric acid and 13.9 M acetic acid, a 60 min heating step (90°C) and elution into toluene overnight according to Jiménez-Bremont et al. (2006) using _L_-proline as a standard (Sigma–Aldrich).

Chlorophyll fluorescence parameter maximum quantum yield of photosystem II photochemistry (*F*_v_/*F*_m_) was determined on dark-adapted plants using a portable fluorometer FluorPen FP100 (PhotonSystems Instruments, Drasov, Czech Republic).

Dehydrin protein relative accumulation was determined using boiling-enriched soluble protein fractions extracted from leaf (L) and crown (C) tissues using 0.1 M Tris-HCl extraction buffer, pH 8.5 with added protease inhibitor (Complete EDTA-free Protease Inhibitor Cocktail Tablets, Roche, Basel, Switzerland). A boiling step was 10 min followed by acetone precipitation. Dry protein pellets were dissolved in Laemmli sample buffer (Bio-Rad, Hercules, CA, USA), concentration of the dissolved proteins was determined using RC DC Protein kit (Bio-Rad) and the samples were loaded on 1D SDS-PAGE gels (12.5% resolving gel) using equal protein loading with respect to protein concentration in plant tissue (fresh weight). For estimation of apparent (electrophoretic) MW of dehydrin proteins, protein marker All Blue Precision Plus Protein Marker Standard (Bio-Rad) was loaded onto the gels. Proteins resolved on 1D SDS-PAGE gel were transferred onto nitrocellulose membrane and were incubated with anti-dehydrin primary antibody raised against dehydrin K-segment (Enzo Life Sciences, Farmingdale, NY, USA) resolved in Tween-20 Tris-buffered saline (TTBS) and then in anti-rabbit alkaline phosphatase and the resulting complexes were visualized using nitroblue tetrazolium/5-bromo-4-chloro-3-indolyl-phosphate (NBT/BCIP) colorimetric detection (Bio-Rad Manual). Immunoblot membranes with visualized dehydrin bands were scanned using a densitometer GS-800 (Bio-Rad) and densitometric analysis of detected dehydrin bands was carried out using Quantity One software, version 4.6.2 (Bio-Rad). Dehydrin band density was calculated relative to a mixed sample containing equal amounts of all samples used in the analysis and loaded on each gel as an internal standard.

Statistical analysis of the data on WSD, ψ_s_, *F*_v_/*F*_m_, proline and dehydrin relative accumulation was performed using STATISTICA, version 12 (StatSoft Inc., Tulsa, OK, USA). Significant differences between the individual experimental variants were evaluated using ANOVA analysis, multiple comparisons, Duncan’s multiple range test (DMRT) at *p* < 0.05.

### SDS-PAGE and In-Gel Digest

Pellets of boiling-soluble proteins-enriched samples for dehydrin analysis extracted from *H. vulgare* and *H. marinum* crowns were dissolved in 50 μl 8 M urea (Sigma–Aldrich, Prague, Czech Republic). Three biological replicates (*n* = 3) were used. The samples were 10 min sonicated (Sonorex, Bandelin, Berlin, Germany) and 1 h incubated at room temperature in thermo shaker (Eppendorf, Říčany, Czech Republic) at 1400 rpm. The sample concentration was determined by BCA Protein Assay (Fisher Scientific, Pardubice, Czech Republic). For an electroforetic separation, the samples were incubated 10 min at 30°C in the thermo shaker and 5 μg of total proteins were loaded on 12% separating gel. The electrophoretic separation (Mini-PROTEAN Tetra Cell, Bio-Rad, Prague, Czech Republic) was carried out for 15 min at 160 V. The gel was stained by Imperial Protein Stain (Fisher Scientific, Pardubice, Czech Republic) according to standard protocol. An in-gel digest was performed according to standard protocol. The protein digestion in bicarbonate buffer by trypsin (Sequencing grade modified trypsin, East Port, Prague, Czech Republic) was carried out for 3 h at 37°C in the thermo shaker. The peptides were extracted by two-stage extraction with mixtures of 35% acetonitrile (AppliChem, Darmstadt, Germany) and 0.1% TFA (Sigma–Aldrich, Prague, Czech Republic) and 70% acetonitrile and 0.1% TFA. The peptides were lyophilized and dissolved in 0.1% TFA prior to a purification by ZipTip C18 (Merck, Prague, Czech Republic). After the purification, the peptides were dried at room temperature.

### NanoLC-ESI-Q-TOF MS

Mass spectrometric analysis was performed using liquid chromatograph UHPL Dionex Ultimate3000 RSLCnano (Dionex, Dreieich, Germany) with mass spectrometer ESI-Q-TOF Maxis Impact (Bruker Daltonics, Bremen, Germany). The lyophilized samples were dissolved in 10 μl of loading buffer (mixture of water:acetonitrile:formic acid in the ratio of 97:3:0,1). Three microliter of the samples were applied to the trap colon Acclaim PepMap 100 C18 (100 μm × 2 cm, reverse phase particle size 5 μm; Dionex, Dreieich, Germany) with a flow rate of 5 μl/min for 5 min. Then, the samples were separated by reverse phase chromatography carried out with a flow rate of 0.3 μl/min through the commercially produced column Acclaim PepMap RSLC C18 (75 μm × 150 mm, reverese phase particle size 2 μm; Dionex, Dreieich, Germany). The separation took place with the following gradient: 0 min 3% B, 5 min 3%B, 85 min 50% B, 86 min 90% B, 95 min 90% B, 96 min 3% B, 110 min 3% B; composition of mobile phase *A* = 0.1% formic acid in water and mobile phase *B* = 0.1% formic acid in acetonitrile. The peptides were eluted directly into the ESI source (Captive spray; Bruker Daltonics, Bremen, Germany). The measurement took place in DDA mode with the selection of precursor in the range of 400–2000 Da. From each MS spectra, up to ten precursors could be fragmented.

### Data Analysis

The peaklists were extracted by Data Analysis 4.1 (Bruker Daltonics, Bremen, Germeny). The proteins were identified by ProteinScape 3 (Bruker Daltonics, Bremen, Germany) with software Mascot 2.4.01 (Matrix Science, London, UK) and the database composed of protein sequences *H. vulgare* contained in the database Uniprot (accessed 30. 01. 2014; 52,296 sequences; 17,742,792 residues belonging to *H. vulgare*) with following parameters: carbamidomethyl (C) as fixed modification, oxidation (M) as variable modification, accuracy 10 ppm in MS mode, MS/MS peptide mass assignment accuracy 0.05 Da and FDR 1%. The proteins which were identified as predicted or uncharacterized were compared to NCBI plant database using BLAST software. Protein domains were determined using PFAM database^1^. Protein functional classification was performed using Gene Ontology database^2^ using the criterion of biological process. The proteins from different samples were compared by Venn diagrams. The proteins which were identified in control and salt-treated samples were quantified by TOP3 methods. This method is based on comparing the average of three most intense peptides of unique protein. This average is directly proportinal to the protein amount in the sample (Ahrné et al., 2013). The ratio of average values and variance of the ratio were calculated using the Taylor series. From the variance was calculated coefficient of variation, which should not exceed 20% (which is a permissible deviation for quantification of proteins based on marking of peptides or proteins). The relevance of quantification was analyzed by Student’s two selection unpaired *t*-test at a level of significance of 0.05 and 0.01 using STATISTICA, version 12.

Cluster analysis on selected 108 proteins present in at least two of the experimental variants was performed using Permut Matrix software, version 1.9.3 (Caraux and Pinloche, 2005). For data analyses, Euclidean distances and Ward’s minimum criteria were used.

## Results

### Physiological Parameters (WSD, PWC, ψ_s_, *F*_v_/*F*_m_, Proline) and Dehydrin Relative Accumulation

**FIGURE 1:**
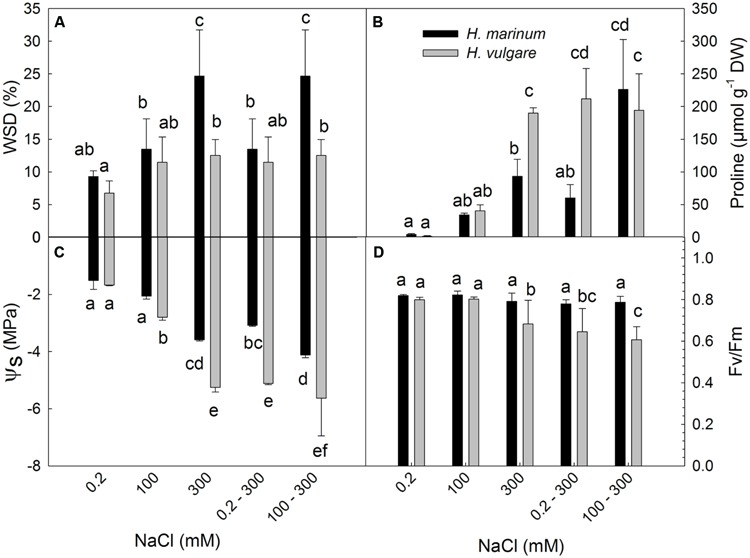
**Water saturation deficit (WSD; A), proline accumulation (B), osmotic potential (ψ_s_; C) and maximum quantum yield of photosystem II photochemistry (*F*_v_/*F*_m_; D) determined in leaf tissues of *Hordeum marinum* (black columns) and *H. vulgare* cv. Tadmor (gray columns)**. Different letters above the columns indicate significant differences between the variants determined by ANOVA, multiple comparisons, Duncan’s multiple range test at 0.05 level.

All salt treatments applied on plants (100; 300; 0.2–300; 100–300) led to an increase in leaf WSD (%), a decrease in osmotic potential of leaf sap (ψ_s_), and an increase in proline levels in both barley species (**Figures 1A–C**). A comparison of both barley species indicated an enhanced increase in WSD while a lower decrease in ψ_s_ in *H. marinum* with respect to *H. vulgare*. Higher WSD levels in *H. marinum* than in *H. vulgare* correspond to lower PWC expressed as ml per 1 g of dry weight in *H. marinum* than in *H. vulgare* which was found not only in plants exposed to high salinity (300 mM NaCl), but also in control plants (Supplementary Figure S2). Only the high salinity treatments (300; 0.2–300; 100–300) led to decreased levels in chlorophyll fluorescence parameter maximum quantum yield of photosystem II efficiency *F*_v_/*F*_m_ in *H. vulgare* while no significant decrease in *F*_v_/*F*_m_ was observed in *H. marinum* (**Figure 1D**).

**FIGURE 2:**
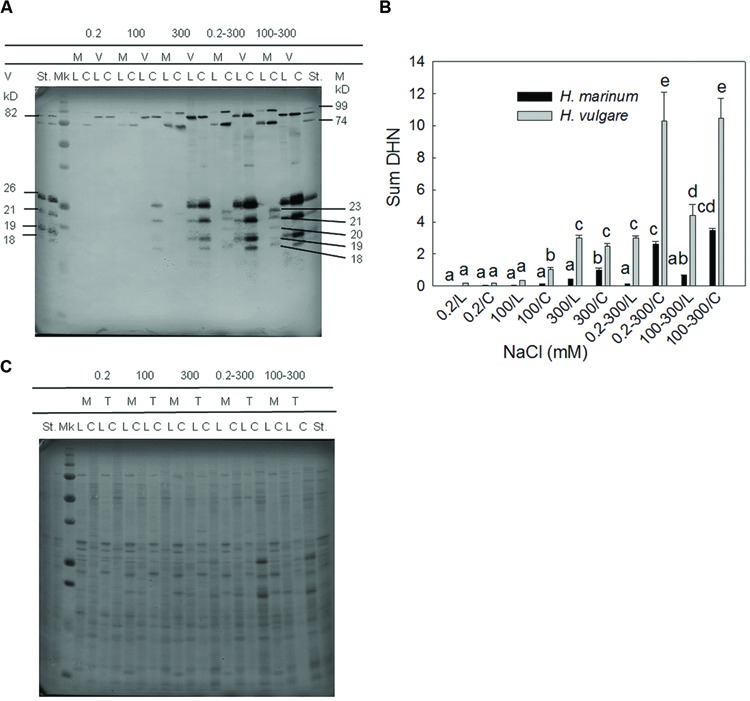
**A representative immunoblot (A) and a graph showing the results of densitometric analysis of dehydrin protein relative accumulation (sum of all dehydrin bands detected in a given sample; B)**. M-*H. marinum* (black columns), V-*H. vulgare* cv. Tadmor (gray columns). C, crown; L, leaf; Mk, protein marker All Blue Precision Plus Protein Marker Standard (Bio-Rad), St - internal standard (a mixed sample containing equal amounts of all samples used in the experiment and loaded on each gel). **(B)** Dehydrin protein relative abundance in the individual samples was expressed as fold changes with respect to the internal standard St. Different letters above the columns indicate significant differences between the variants determined by ANOVA, multiple comparisons, Duncan’s multiple range test at 0.05 level. **(C)** Proteins transblotted onto nitrocellulose membrane were visualized by staining with Ponceau S (Sigma–Aldrich).

All salinity treatments led to an accumulation of dehydrin proteins in both barley genotypes (**Figure 2**). Both quantitative and qualitative differences were found between both barley species. The level of dehydrin protein relative accumulation in *H. marinum* was significantly lower than in Tadmor under the same treatment. Both barley species accumulated high-molecular and low-molecular dehydrin proteins in response to salinity; however, the number and position of detected dehydrin bands were different between the species. *H. vulgare* cv. Tadmor accumulated one high-molecular dehydrin band of 82 kD corresponding to DHN5 and four low-molecular dehydrin bands of 26, 21, 19, and 18 kD in accordance with our previous results (Kosová et al., 2015). In contrast, *H. marinum* reveals two high-molecular dehydrin bands of apparent MW 99 and 74 kD and five low-molecular dehydrin bands of apparent MW 23, 21, 20, 19, and 18 kD (**Figure 2A**). However, in *H. marinum*, the low-molecular-weight dehydrins were detected only in the samples transferred directly from control to high salinity (0.2–300 mM NaCl) while they were absent in the samples gradually acclimated to 300 mM NaCl. In both species, a significantly higher dehydrin accumulation was found in crown tissues with respect to leaf tissues (**Figures 2A,B**).

### Proteomic Analysis

**FIGURE 3:**
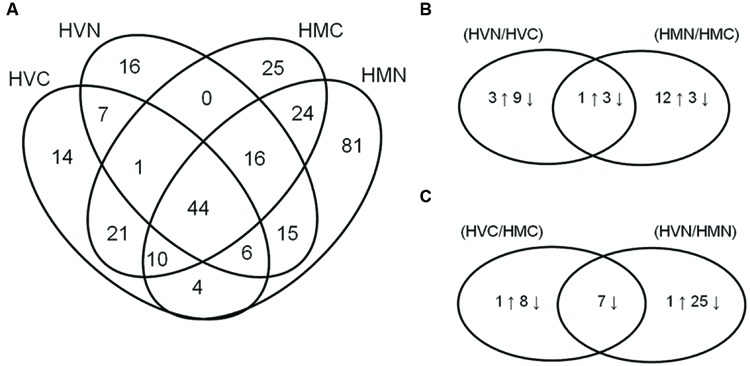
**Venn diagrams showing the number of identified proteins in all experimental variants studied (A; HVC, *Hordeum vulgare*, control treatment; HVN, *H. vulgare*, 300 mM NaCl; HMC, *H. marinum*, control treatment; HMN, *H. marinum*, 300 mM NaCl) and Venn diagrams showing identified proteins revealing significant differences in their relative protein abundance between control and salt-treated plants **(B)** and between *H. vulgare* and *H. marinum* genotypes **(C)****. **(B,C)** the number of proteins showing significant differences between the experimental variants is indicated. Differences were evaluated by unpaired Student’s *t*-test at 0.05 level.

Crown tissues of two barley species *H. vulgare* (HV-) and *H. marinum* (HM-), both control (0.2 mM NaCl; HVC, HMC) and salt-treated samples (300 mM NaCl; HVN, HMN) after the one-step transfer to high salinity (0.2–300 mM NaCl), were used for proteomic analysis. Three biological replicates (*n* = 3) of each sample were used for the analysis. Proteins which remain soluble upon a short boiling step of 10 min. were separated by 1D SDS-PAGE gels which were divided into four fractions according to protein electrophoretic molecular weight for protein identification (Supplementary Figure S3). In total, 284 differential proteins were identified by LC-MS (ESI-Q-TOF) in the four experimental variants (**Figure 3**; Supplementary Tables S1 and S2). In *H. vulgare* control plants (0.2 mM NaCl; HVC), 107 proteins were identified, in *H. vulgare* salt-treated plants (300 mM NaCl; HVN), 105 proteins were identified, in *H. marinum* control plants (0.2 mM NaCl; HMC), 141 proteins were identified, and in *H. marinum* salt-treated plants (300 mM NaCl; HMN), 200 proteins were identified (**Figure 3A**). A comparison of the proteins identified in both control and salt-treated plants has revealed that *H. vulgare* exhibited a higher number of proteins decreased under high salinity with respect to control than proteins increased in response to high salinity (4↑12↓); in contrast, *H. marinum* revealed a higher number of proteins increased in response to salinity with respect to the number of decreased proteins (13↑6↓; **Figure 3B**). Similarly, a relatively high number of proteins (25) revealed a decreased relative abundance in salt-treated *H. vulgare* than in salt-treated *H. marinum* (**Figure 3C**). A list of identified proteins belonging to the individual groups in Venn diagrams is given in Supplementary Table S3.

**FIGURE 4:**
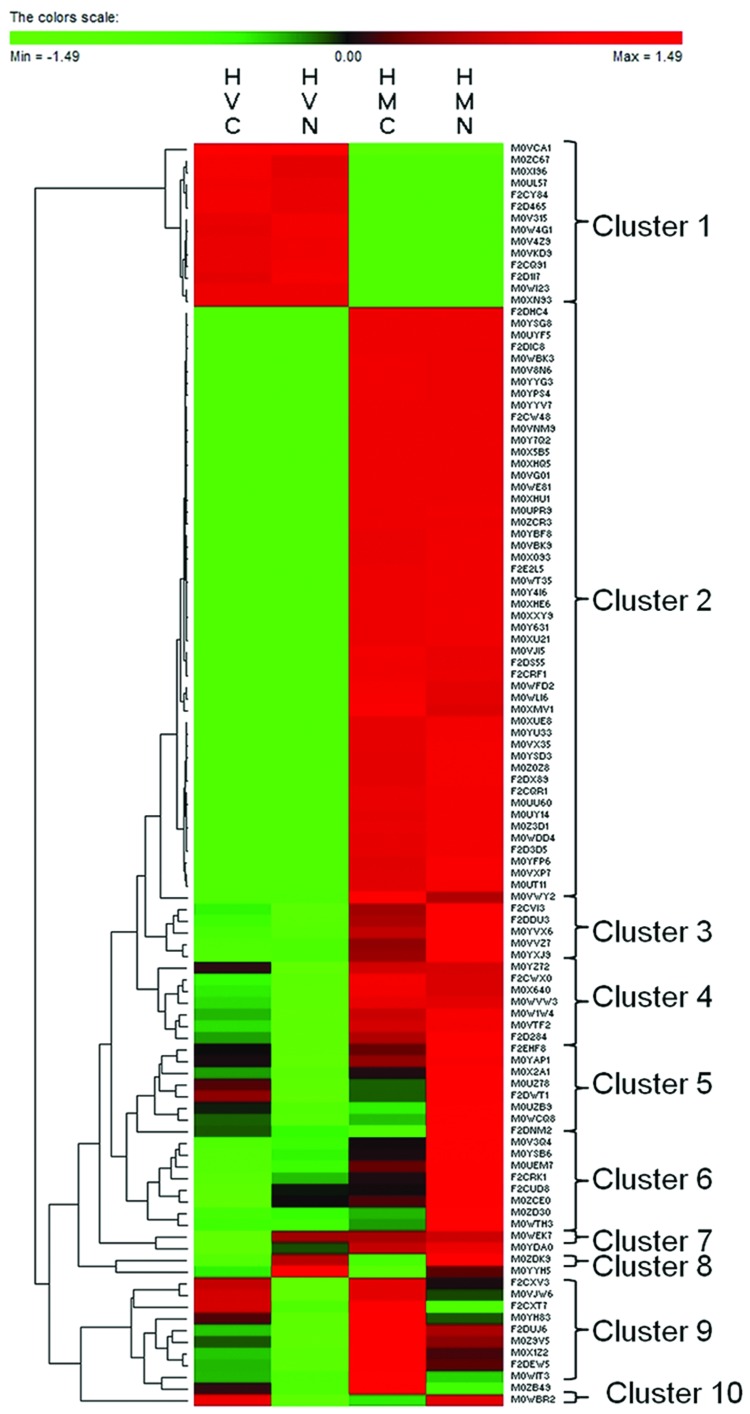
**Cluster analysis of 108 proteins identified in at least two experimental variants except for the variants HVC ∩ HMC (21), HVN ∩ HMN (15), HVC ∩ HMN (4), and HVN ∩ HMC (0)**. HVC, *H. vulgare*, control treatment; HVN, *H. vulgare*, 300 mM NaCl; HMC, *H. marinum*, control treatment; HMN, *H. marinum*, 300 mM NaCl.

Out of 284 proteins identified in all four variants, 136 proteins were identified in one variant only while 148 proteins were identified in at least two experimental variants. Cluster analysis was carried out for 108 proteins present in at least two experimental variants except for the proteins found in HVC ∩ HMC (21), HVN ∩ HMN (15), HVC ∩ HMN (4), and HVN ∩ HMC (0) only. Cluster analysis has distinguished 10 clusters regarding protein dynamics between the variants (**Figure 4**). Cluster 1 (14 proteins) includes proteins identified only in *H. vulgare* while cluster 2 (51 proteins) includes proteins identified only in *H. marinum*. Cluster 3 (five proteins) and cluster 4 (seven proteins) include proteins with an increased relative abundance in *H. marinum* with respect to *H. vulgare*. Cluster 5 (eightproteins) includes proteins with relatively enhanced abundance in HVC and HMN with respect to HVN and HMC variants. Cluster 6 (eight proteins) includes proteins with relatively increasing pattern from HVC via HVN and HMC to HMN, i.e., proteins with the highest abundance in HMN variant while the lowest abundance in HVC variant. Cluster 7 (2 proteins) and cluster 9 (10 proteins) include proteins with relatively enhanced abundance in salt-treated variants HVN, HMN) with respect to control variants (HVC, HMC) while cluster 8 (two proteins) includes proteins with relatively enhanced abundance in salt-treated variants (HVN, HMN) than in control samples (HVC, HMC). Cluster 10 (one protein) reveals relatively higher abundance in HVC and HMN than in HVN and HMC. The list of identified proteins belonging to the individual clusters is given in Supplementary Table S4.

**FIGURE 5:**
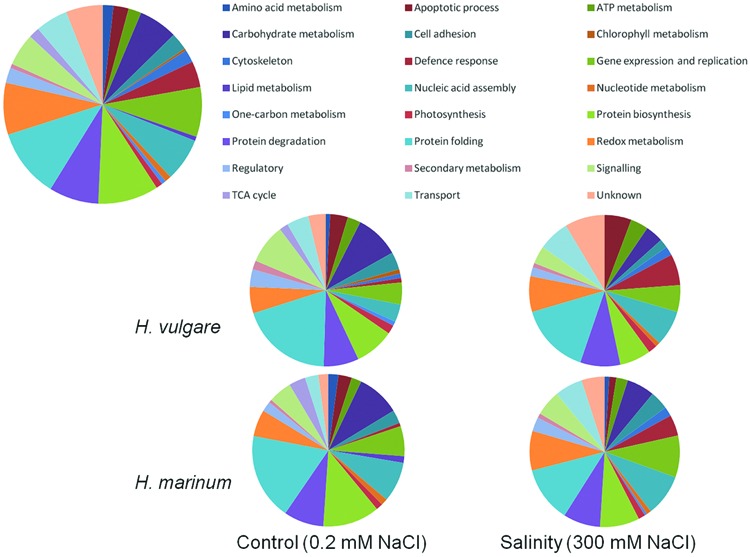
**Functional classification of identified proteins according to biological process**. The upper diagram shows functional classification of all 284 identified proteins in the experiment while the four lower diagrams show functional classification of the identified proteins in the individual experimental variants.

The 284 identified proteins were classified into 24 functional groups based on Gene Ontology classification of biological processes: amino acid metabolism (5 proteins), apoptotic process (7 proteins), ATP metabolism (6 proteins), carbohydrate metabolism (18 proteins), cell adhesion (8 proteins), chlorophyll metabolism (1 protein), cytoskeleton (6 proteins), defense response (12 proteins), gene expression and replication (23 proteins), lipid metabolism (2 proteins), nucleic acid assembly (20 proteins), nucleic acid metabolism (3 proteins), one-carbon metabolism (2 proteins), photosynthesis (3 proteins), protein biosynthesis (28 proteins), protein degradation (23 proteins), protein folding (32 proteins), redox metabolism (24 proteins), regulatory proteins (7 proteins), secondary metabolism (2 proteins), proteins involved in signaling (15 proteins), proteins of tricarboxylic acid cycle (5 proteins), proteins involved in cellular transport (15 proteins), and proteins of unknown function (17 proteins). Several proteins were identified as predicted proteins or uncharacterised proteins and their possible identity was determined using BLAST search against Uniprot database (downloaded January 30, 2014). An overview of all 284 proteins identified in the experiment ordered according to their biological functions is given in **Figure 5**; Supplementary Tables S1 and S5.

## Discussion

### Plant Physiological Response and Dehydrin Relative Accumulation

All salt treatments applied on plants (100; 300; 0.2–300; 100–300) indicated a significant dehydration effect of salinity on both genotypes. A comparison of the two barley species has shown higher leaf tissue dehydration in *H. marinum* with respect to *H. vulgare* while a lower decrease in ψ_s_ associated with an enhanced accumulation of both low-molecular osmolytes and osmotically active proteins (dehydrins) in *H. vulgare* which may compensate enhanced Na^+^ accumulation in *H. vulgare* shoot vacuoles as shown in a previous study (Garthwaite et al., 2005). Higher WSD levels in *H. marinum* than in *H. vulgare* correspond to lower PWC in *H. marinum* than in *H. vulgare* which was found not only in plants exposed to high salinity (300 mM NaCl), but also in control plants (Supplementary Figure S1). These data are in accordance with the previously found results (Garthwaite et al., 2005) and indicate constitutive differences in shoot water relations between *H. marinum* and *H. vulgare* which may be associated with differential levels of Na^+^ ions in shoot tissues.

Regarding dehydrins, both quantitative and qualitative differences in dehydrin accumulation under salinity treatments indicate differential roles of dehydrins in salt-treated *H. marinum* and *H. vulgare*, respectively. Higher dehydrin levels found in crowns than in leaves correspond to the crucial role of crown tissues for the whole plant survival. Similarly, an enhanced dehydrin accumulation was found in cold-treated crown tissues of winter wheat (Houde et al., 1995). One-step transfers from control and low-salinity conditions to high-salinity conditions resulted in higher accumulation of dehydrin proteins with respect to acclimation to gradually increasing salt levels, especially in salt-tolerant *H. marinum*. This corresponds to the results of our previous study (Kosová et al., 2015). Crowns are also closer to root salt ion uptake than leaves so it can be expected that they contain higher Na^+^ levels. Thus, increased dehydrin accumulation in crown cell cytoplasm may help to compensate increased Na^+^ levels in crown vacuoles.

Only the high salt treatments (300; 0.2–300; 100–300) led to a significant decrease in *F*_v_/*F*_m_ levels indicating an adverse stress impact of salinity on the efficiency of photosynthetic processes (**Figure 1D**). Under optimum conditions, *F*_v_/*F*_m_ values range around 0.83 (Lichtenthaler and Rinderle, 1988); however, severe stress leads to a decrease in *F*_v_/*F*_m_ values in sensitive plants indicating a reduced energy trapping efficiency by damaged photosystem II reaction centre (RC PSII), partially due to a damaged OEC (Rizza et al., 2011; Athar et al., 2015). Decreased *F*_v_/*F*_m_ values in *H. vulgare* cv. Tadmor exposed to high salt stress (300 mM NaCl) were also found in our previous study (Kosová et al., 2015). Significantly decreased *F*_v_/*F*_m_ levels in leaf tissues of *H. vulgare* cv. Tadmor exposed to high salinity levels (300; 0.2–300 and 100–300 mM NaCl treatments) indicate impairments in primary photosynthetic processes of salt-affected plants. Similar results were found in other salt-sensitive plants such as rice (Kim et al., 2005). In contrast, *F*_v_/*F*_m_ values in leaf tissues of *H. marinum* remained unchanged under high salinity with respect to control plants indicating no significant adverse effect of salinity on the efficiency of primary photosynthetic processes. The observed differences in *F*_v_/*F*_m_ between *H. vulgare* and *H. marinum* exposed to high salinity treatments indicate differential response of photosynthetic apparatus to salinity in these two species and they are in accordance with differential levels of OEE proteins and several chloroplast-located chaperones found by proteomic analysis (see below).

The differences between the two barley genotypes observed in tissue dehydration (WSD, PWC, and ψ_s_) and osmolyte accumulation (proline, dehydrins) are in accordance with the previously published data (Garthwaite et al., 2005; Islam et al., 2007). These studies have reported differential levels of salt ions and differential mechanisms of salt tolerance in *H. marinum* and *H. vulgare*, respectively. Whereas *H. marinum* reveals low Na^+^ levels in the shoots under salinity due to efficient Na^+^ exclusion at xylem level, *H. vulgare* seems to employ Na^+^ vacuolar compartmentation which is compensated by enhanced accumulation of osmotically active compounds such as proline and dehydrin proteins in cell cytoplasm. Therefore, higher levels of proline and sum of dehydrin proteins as well as lower levels of osmotic potential found in *H. vulgare* cv. Tadmor with respect to *H. marinum* are in accordance with the observed differences in salinity response between the two barley species.

### Proteomic Analysis

The results of physiological analyses including characteristics related to tissue dehydration (WSD, PWC, and ψ_s_) and osmolyte accumulation (proline, dehydrins) as well as efficiency of photosynthetic apparatus (*F*_v_/*F*_m_) led us to selection of one-step direct transfer to high salinity (0–300 mM NaCl) for further proteomic study since this treatment induced a significant stress response in both *H. vulgare* and *H. marinum*. For example, only the one-step direct transfers to high salinity induced an accumulation of low-molecular weight dehydrins in *H. marinum* (**Figure 2A**). Crown tissues were used for protein isolation since crowns are crucial for whole plant survival in cereals. We decided to use the boiling step commonly applied for dehydrin isolation for sample enrichment with several stress-responsive proteins and chaperones.

An analysis of 1D SDS-PAGE protein fractions has led to an identification of a total of 284 proteins in both barley genotypes under control and high salinity treatments (**Figure 3A**). A comparison of both barley species in Venn diagrams has revealed more proteins enhanced in salt-tolerant *H. marinum* with respect to *H. vulgare* under both control (15↑) and high salinity (32↑) conditions (**Figure 3C**). These results are in accordance with those reported by Pang et al. (2010) who found constitutively enhanced levels of several stress-responsive proteins in salt-tolerant *T. halophila* with respect to salt-sensitive *A. thaliana*.

A comparison of control variants versus high salinity variants based on protein functional categories (**Figure 5**) revealed that salt-treated samples with respect to control ones exhibited a higher percentage of identified proteins involved in defense response (HVN 6.7%, HMN 4.5%, HVC 0.93, and HMC 0.7% of total identified proteins in the given experimental variant) and in redox metabolism (HVN 7.6%, HMN 8.5%, HVC 5.6%, and HMC 5.7% of total identified proteins in the given experimental variant). In contrast, control samples with respect to salt-treated ones revealed higher percentage of identified proteins involved in carbohydrate metabolism (HVC 9.3%, HMC 9.2%, HVN 3.8%, and HMN 6% of total identified proteins in the given experimental variant) and protein biosynthesis (HVC 8.4%, HVN 6.7%, HMC 12%, and HMN 8.5% of total identified proteins in the given experimental variant). Salt-treated *H. vulgare* (HVN) revealed relatively higher percentage of proteins involved in apoptotic processes (5.7% of total identified proteins) with respect to other variants (1.5–3.7% of total identified proteins in the individual variants; **Figure 5**; Supplementary Table S5).

The presence of several boiling-unstable proteins in a boiling-soluble protein fraction can be explained by presence of several chaperones and other proteins with protective functions which can protect other proteins from a lack of their conformation and precipitation. The identification of several ubiquitious proteins such as enzymes of tricarboxylic acid cycle and several structural proteins such as transmembrane channels (e.g., components of mitochondrial outer and inner envelope complexes) only in some experimental variants can be explained by differential relative abundance of these proteins with respect to other proteins in the whole protein sample which can be under detection threshold in some samples as well as by strict identification criteria (a minimum of two matched peptides) which could not be fulfilled in all samples.

### Possible Roles of Identified Proteins in *H. marinum* and *H. vulgare* Salinity Response with Respect to Their Functional Classification

#### Signaling and Signal Transduction

Proteins revealing significant quantitative or qualitative differences between experimental variabnts included PP2C, small calcium-binding proteins and 14-3-3 proteins (**Figure 5**; Supplementary Table S1).

Protein phosphatase 2C is known to act antagonistically to MAPKKK kinase cascade and SnRK involved in ABA-mediated signaling and signal transduction from plasmalemma to nucleus. It is known that ABA receptor PYR1 activated by ABA inhibits PP2C (Park et al., 2009). A decrease in PP2C 10 (M0Z9V5) levels under salinity with respect to control plants may indicate alterations in regulation of ABA-mediated signaling pathways under salinity stress with respect to control conditions. Differential phosphorylation of PP2C under drought with respect to control conditions was found in wheat (Zhang et al., 2014).

Three small calcium-binding proteins, calcium-binding protein CML7 (F2E2L5), calmodulin (F2CQ91) and calreticulin (F2CWX0) and one protein with an IQ calmodulin-binding motif (M0WVJ8) were identified in the experiment. An increase in calmodulin levels was found in HVN with respect to HVC, while decreased levels of calmodulin and calreticulin were found in HVN with respect to HMN. Small calcium-binding proteins are known to play an important role in stress-induced signaling as second messengers transducing the original signal via interactions with several kinases and phosphatases. Increased levels of calmodulin and calreticulin were found under salinity in *Arabidopsis* roots (Jiang et al., 2007), in rice roots (Cheng et al., 2009), in grapevine shoots (Vincent et al., 2007), and others.

Three 14-3-3 proteins (14-3-3A F2CRF1; 14-3-3B M0XMV1; 14-3-3E A1X810) were identified in three experimental variants except HVN. 14-3-3 proteins contain a TPR motif and they are known to be involved in modulation of cell signaling, interactions with other proteins and plant-pathogen interactions. They can also regulate activity of vacuolar H^+^-ATPases thus affecting Na^+^ vacuolar sequestration (Finnie et al., 1999). Some 14-3-3 proteins can be found even in extracellular space where they interact with proteins produced by plant pathogens (Denison et al., 2011). A decrease in 14-3-3 like protein B level was found in HMN with respect to HMC which is consistent with the results observed in salt-tolerant *Puccinellia tenuiflora* (Yu et al., 2011); in contrast, an increase in 14-3-3 protein was found in salt-treated *Kandelia candel* (Wang et al., 2014).

#### Gene Expression and Nucleic Acid Assembly

Nascent associated-polypeptide complex (NAC α) proteins are TFs involved in both ABA-dependent and ABA-independent pathways which bind to NACR *cis*-regulatory elements and regulate alone or in cooperation with zinc-finger homeodomain TFs (ZFHD TFs) expression of several dehydration-responsive genes such as *RD22* in *A. thaliana* (Yamaguchi-Shinozaki and Shinozaki, 2006; Puranik et al., 2012). In our study, three NAC proteins (F2D1I7, M0VX35, and M0YAP1) were identified in both salt-treated barleys with HMN revealing significantly higher abundance of NAC proteins than HVN. Enhanced levels of NAC α were also reported in salt-treated rice roots (Yan et al., 2005) indicating the importance of NAC α in regulation of salt-responsive protein pathways. An identification of a bZIP (M0Z1P5) in HMN may indicate an enhanced expression of several ABA-inducible stress-responsive genes including several LEA proteins (Choi et al., 1999).

Three proteins (M0ZC67, M0ZC68, and M0XUE8) identified as nucleosome assembly protein 1-A as well as protein SET (M0VVZ7) are involved in interaction with nucleosomal histones thus affecting the dynamics of euchromatin-heterochromatin transitions and DNA accessibility for transcription. Nucleosome assembly proteins also participate on transcriptome reprogramming in plants exposed to altered environmental conditions as reported for cold-treated winter barley (Janská et al., 2011). Decreased levels of SET protein were found in *H. vulgare* with respect to *H. marinum* under both control and salinity conditions. DNA repair protein RAD23 (M0YFP6, M0Z6M4, and F2DUJ6) is involved in nucleotide excision and repair (GO:0006289). However, RAD23 is also known to inhibit a polyubiquitin chain formation and 26S proteasome-dependent degradation of proteolytic substrates in yeast thus linking DNA repair to ubiquitin/proteasome pathway (Schauber et al., 1998).

#### Protein Metabolism

##### Protein biosynthesis

Several proteins involved in protein biosynthesis were identified including both cytosolic and organellar ribosomal proteins and translation initiation and elongation factors. Some of them were detected only in control variants (HVC, HMC; 40S ribosomal protein SA M0WYK5; 60S acidic ribosomal protein P0 F2DBD4; 60S ribosomal protein L22-2 F2EAX5; organellar elongation factor Tu M0ZD98) while others were detected only in *H. marinum* (HMC, HMN) variants (elongation factor 1 β M0YVB7; elongation factor 1 γ 2 M0WF40; eukaryotic translation initiation factor 5A2 M0YU33; 40S ribosomal protein S12 M0VTY9; 40S ribosomal protein S19 F2E598; 30S ribosomal protein 2 M0XX22). Several ribosomal proteins reveal specific functions in translation regulation; for example, 60S acidic ribosomal proteins (60S acidic ribosomal protein P2B M0V315; 60S acidic ribosomal protein P0 F2DBD4; 60S acidic ribosomal protein P2 M0YSB6; 60S acidic ribosomal protein P3 M0XHQ5) are known to be regulated by phosphorylation and they are involved in interaction with elongation factor EF1. A decrease in 60S acidic ribosomal protein P2 (M0YSB6) was found in HVN with respect to HMN. The 30S ribosomal protein S1 is important for translation initiation due to mRNA recognition and binding to mRNA upstream of Shine–Dalgarno sequence (Boni et al., 1991). An enhanced relative abundance of 30S ribosomal protein S1 (M0YSG8) found in *H. marinum* with respect to *H. vulgare* under control conditions indicates possible alterations in organellar proteosynthesis between the two barley species. Identification of several ribosomal proteins and translation initiation and elongation factors indicates active protein biosynthetic processes which are crucial for both control and stress-treated plants to underlie an active stress acclimation. 60S ribosomal protein L12 and 50S ribosomal protein L12-2, respectively, are known to bind to rRNA during ribosome assembly and are involved in maintenance of ribosomal structure and function. In our experiment, these proteins were detected in salt-treated plants of both species (HVN, HMN) and in *H. marinum*, respectively. Alterations in the levels of 60S acidic ribosomal protein P1, 30S ribosomal protein S1 and ribosomal protein L12 homolog were found in salt-treated barley leaves (Fatehi et al., 2012). An enhanced abundance of ribosomal protein L12 was reported in salt-treated rice leaves (Kim et al., 2005).

Some translation initiation and elongation factors reveal multiple functions besides regulation of protein synthesis. Eukaryotic translation initiation factor 5A (eIF-5A) is known to be activated by PTM of hypusination and it is involved in regulation of cell cycle. It is also known that eIF-5A isoforms differing in hypusination level play differential roles in cell cycle regulation and cell fate determination with eIF-5A1 isoform involved in induction of cell apoptosis while eIF-5A2 isoform involved in induction of cell division (Thompson et al., 2004). In our experiment, eIF-5A1 isoform was found in *H. vulgare* while eIF-5A2 isoform was found in *H. marinum* which is consistent with the presence of several proteins involved in apoptosis induction in salt-treated *H. vulgare* while their absence in *H. marinum*.

##### Protein degradation

Proteins involved in protein ubiquitination resulting in protein targeting for proteasome degradation (ubiquitin-like protein M0V112; E3 UMF1-protein ligase 1 homolog M0W4G1; deubiquitination-protection protein dph1 F2CQR1; small ubiquitin-related modifier 1 M0UGE2) are increased in salt-treated variants (HVN, HMN) with respect to control ones (HVC, HMC) indicating enhanced protein damage under salinity. In addition, 26S protease regulatory subunit 6A-like protein A (M0ZB49) reveals relatively enhanced level in HVN with respect to HMN indicating relatively enhanced proteasome-dependent protein degradation in HVN with respect to HMN. 26S protease regulatory subunit 6A-like protein A reveals ATPase activity and contains AAA^+^-ATPase domain. AAA^+^-ATPases are present in all living organisms and reveal a wide array of functions including facilitation of protein folding and defolding, assembly and disassembly of protein complexes, and proteolysis. Enhanced levels of proteins involved in proteasome-dependent protein degradation were reported in salt-treated *Nitraria sphaerocarpa* (Chen et al., 2012).

Four proteins with cysteine proteinase activity (papain-like cysteine proteinase B4ESE6, M0ZD30; thiol protease aleurain M0VNM9; triticain β 2 M0YY63) and three cystatins (M0V101, F2CUF5, and F2DNM2), cysteine proteinase inhibitors, were identified in the samples, predominantly in HMN. In HMN, relatively enhanced levels of papain-like cysteine protease and cystatin Hv-CPI14 with respect to control indicate an activation of stress-responsive protein protective mechanisms under salinity. Cystatins are known to respond to pathogen attack via the inhibition of pathogen cysteine proteinases (Benchabane et al., 2010). They could be induced also by various abiotic stresses including cold (Kosová et al., 2013b) and salinity, as seen in the present study.

#### Energy Metabolism

##### Photosynthesis

Three OEE proteins involved in formation of OEC, a crucial component of photosystem II (PSII) where photolysis of water occurs, were identified (OEE1 F2CRK1; OEE2 M0WTH3; OEE3-1 M0YTD9). Two of them (OEE1, OEE2) were present in all four experimental variants and revealed a significant increase in salt-treated *H. marinum* with respect to control plants as well as enhanced levels in *H. marinum* with respect to *H. vulgare*, especially in salt-treated variants (HVN, HMN). Relatively enhanced abundance of OEE1 and OEE2 proteins in *H. marinum* with respect to *H. vulgare*, especially under high salt treatment, indicates an active stress acclimation of *H. marinum* when compared to *H. vulgare*. Enhanced levels of some PSII components such as D2 protein, a crucial component of RC PSII, and OEE1 and OEE2 proteins, under salinity were found in tolerant halophytic species such as *Suaeda aegyptiaca* (Askari et al., 2006) and *Aeluropus lagopoides* (Sobhanian et al., 2010), respectively; in contrast, a decrease in PSII protein components was found in salt-sensitive species such as durum wheat (Caruso et al., 2008).

##### ATP metabolism

Six proteins identified include proteins involved in ATP biosynthesis such as mitochondrial ATP synthase precursor as well as proteins involved in cleavage or transfer of phosphate groups resulting in interconversion of nucleotide phosphates (adenosine kinase 2 M0XLC4; adenylate kinase A F2DTH4; nucleoside diphosphate kinase F2CXV3; soluble inorganic pyrophosphatase 1 F2EJL5, F2CVI3). ATP metabolism plays a crucial role in plant stress acclimation since an active stress acclimation process requires a large portion of energy. Soluble inorganic pyrophosphatase is involved in cleavage of macroergic phosphate bonds in pyrophosphate to yield single phosphates. Tonoplast-bound inorganic pyrophosphatase is involved in Na^+^/H^+^ antiport across tonoplast and was found enhanced upon salt stress in both glycophytes and halophytes (Jiang et al., 2007; Wang et al., 2008; Du et al., 2010; Pang et al., 2010; Xu et al., 2010). Two proteins (F2EJL5, F2CVI3) identified as soluble inorganic pyrophosphatase 1 reveal a decreased abundance in HVN with respect to HMN indicating differential energy requirements. The presence of mitochondrial ATP synthase precursor (M0XMC3) in salt-treated variants (HVN, HMN) corresponds with enhanced need for energy during an active stress acclimation as well as with the results of other proteomic studies on halophytes *A. lagopoides* (Sobhanian et al., 2010) and *Tangut nitraria* (Cheng et al., 2015).

##### Tricarboxylic acid (TCA) cycle

Five proteins belonging to TCA cycle (aconitate hydratase M0Y4D4; isocitrate dehydrogenase M0YIA7; succinyl-CoA ligase subunit β F2D1J8; malate dehydrogenase M0Z0D3, F2CQR0) were detected only in control plants (HVC, HMC); all of them were identified in *H. marinum* while two of them in *H. vulgare* (Supplementary Tables S1 and S5). Detection of TCA cycle enzymes only in control plants may indicate an enhanced abundance of these enzymes in non-stressed plant materials with respect to salt-treated ones. Moreover, higher levels of several protein chaperones in *H. marinum* with respect to *H. vulgare* may contributed to the preservation of relatively larger amounts of TCA cycle enzymes in HMC with respect to HVC during sample preparation. Similarly, a relatively enhanced level of malate dehydrogenase was found in high frost-tolerant winter wheat Mironovskaya 808 with respect to less frost-tolerant winter wheat Bezostaya 1 (Vítámvás et al., 2012).

#### Carbohydrate Metabolism

##### Catabolic enzymes

In the samples, six out of twelve glycolytic enzymes were identified including fructose bisphosphate aldolase (ALDO; F2CXT7), triosephosphate isomerase (TPI; F2EHF8, M0WDD4), glyceraldehyde-3-phosphate dehydrogenase 2 (GAPDH; F2D6I8), phosphoglycerate kinase (PGK; M0Y9H9), PGM (M0WLI6), and ENO (M0WLI6). A significant decrease in relative abundance of four glycolytic proteins detected (ALDO, ENO, PGM, and TPI) was found in salt-treated samples with respect to control ones, especially in *H. vulgare*, indicating a downregulation of crucial metabolic pathways under severe stress. In halophytes such as *Bruguiera gymnorhiza* and *Halogeton glomeratus*, enhanced levels of several glycolytic enzymes such as ALDO and GAPDH were found (Tada and Kashimura, 2009; Wang et al., 2015). An induction of several glycolytic enzymes (GAPDH, TPI) was found in salt-treated durum wheat (Caruso et al., 2008); however, the plants were exposed to 100 mM NaCl indicating a mild stress treatment only. A decrease in glycolytic enzymes levels in HVN indicates a severe stress treatment; as found in our previous study on spring barley Amulet exposed to two intensities of drought, a mild stress usually leads to enhanced levels of glycolytic enzymes indicating an active plant stress acclimation while a more severe stress can result in a decrease in glycolytic enzymes indicating a disruption of cellular metabolism (Vítámvás et al., 2015). It can be concluded that high salt treatment induced an active acclimation in *H. marinum* while damage in *H. vulgare*.

##### Anabolic enzymes

Phosphoglycerate kinase involved in gluconeogenesis and transaldolase involved in pentose phosphate pathway were found in control variants of both barley species which may indicate relatively enhanced need for hexoses and pentoses in control plants with respect to salt-treated ones. Sucrose synthase catalyzes a reversible cleavage of sucrose to yield fructose and UDP-glucose which can be then incorporated into polysaccharide chains. The identification of sucrose synthase (M0UKI5) in control variants (HVC, HMC) only may indicate enhanced glycosidic bond formation in control variants which corresponds to an enhanced need for energy, i.e., rather a cleavage than a formation of glycosidic bonds, in salt-treated variants. Several enzymes involved in modification of cell wall polysaccharides such as UDP-glucuronate decarboxylase (F2D5Z0), UTP-glucose-1-phosphate uridylyltransferase (M0USZ9) were found only in control plants while endo-1,3:1,4-β-_D_-glucanase (F2DSV5) was found only in HMN variant. Protein identified as invertase inhibitor (M0W0Q7) in HMN variant is known to bind to invertases and pectinesterases inhibiting their activity thus affecting cell wall elongation and fruit ripening. These results indicate profound alterations in composition of cell wall polysaccharides in salt-treated plants with respect to controls, especially in HMN, which corresponds to the results of Mostek et al. (2015).

#### Other Metabolic Pathways

##### Amino Acid Metabolism

Five proteins involved in amino acid metabolism were identified, four of them were identified only in *H. marinum* (both control and salt-treated samples). Tryptophan aminotransferase 1 (F2E3H9), aspartate aminotransferase (M0USC9) are involved in transfer of amino groups resulting in interconversion between aminoacids and oxoacids. Cysteine synthase (M0VBS3) is involved in biosynthesis of cysteine while serine hydroxymethyltransferase (F2DET3) is involved in interconversion between glycine and serine. Amino acids seem to play a protective role under salinity due to their osmoprotectant and ROS scavenging effects (Sobhanian et al., 2010; Flowers and Colmer, 2015).

##### Chlorophyll metabolism

One protein was identified as magnesium chelatase which is an enzyme catalyzing ATP-dependent incorporation of Mg^2+^ into protoporphyrin IX during chlorophyll biosynthesis. This protein was found only in HVC. Magnesium chelatase was found decreased in salt-treated halophyte *Tangut nitraria* (Cheng et al., 2015).

##### Lipid metabolism

Two enzymes, ENR (M0YUL2) and GDSL esterase/lipase (M0VZB7), were identified only in HMC. ENR is a component of fatty acid synthetase type II in plastids and it is involved in a reduction step during fatty acid chain biosynthesis in thylakoid membranes which is crucial for thylakoid membrane integrity upon salt stress. The presence of ENR in HMC may indicate constitutive protection of thylakoid membranes in *H. marinum*. Changes in ENR levels were also found in salt-treated rice panicles (Dooki et al., 2006).

##### One-carbon metabolism

Two enzymes associated with one carbon metabolism were identified. *S*-adenosylhomocysteine hydrolase (M0ZB73) cleaves *S*-adenosylhomocysteine, a product after the methyl transfer from SAM, into adenosine and homocysteine. Cyanate hydratase, also known as cyanase, is involved in detoxification of cyanate since it catalyzes a conversion of cyanate to carbamate which is then spontaneously decomposed into carbon dioxide and ammonia. Cyanate in plants arises as a by-product during ethylene biosynthesis. Cyanate hydratase (M0UZA5) was identified only in HMN. Similarly, cyanate hydratase was identified in salt-treated halophyte *S. aegyptiaca* by Askari et al. (2006) and the authors speculated about its role not only in detoxifying cyanate, but also in supplying salt-treated plants with alternative nitrogen and carbon sources. Thus, the presence of cyanate hydratase in HMN may underlie its superior salt tolerance under high salt stress.

#### Stress and Defense-Related Proteins

##### Protein folding

Several proteins involved in protein folding revealed enhanced levels in salt-treated plants with respect to control ones as well as in salt-treated *H. marinum* with respect to *H. vulgare* (M0X093 luminal binding protein 4; M0WRW4, F2DA67, M0WCQ8 PPI; M0UZ78, F2DU3 20 kDa chaperonin; M0X640 RubisCO large subunit-binding protein subunit β; M0Y631, M0V4Z9 chaperonin CPN60-2; M0UEM7 HSP STI; M0WVW3, M0VTF2 stromal 70 kDa heat shock-related protein), out of which, a substantial amount represent chloroplast-located proteins involved in protection of photosynthetic apparatus and RubisCO carbon assimilation activity (luminal binding protein 4; RubisCO large subunit-binding protein subunit α and β, stromal 70 kDa heat shock-related protein, chaperonin CPN 60-2) indicating enhanced protection of photosynthetic apparatus in halophytic *H. marinum* with respect to glycophytic *H. vulgare*. An increase in chloroplast RubisCO large subunit-binding protein subunit β was found in salt-sensitive durum wheat (Caruso et al., 2008) and barley (Rasoulnia et al., 2011; Fatehi et al., 2012) as well as in salt-tolerant *P. tenuiflora* (Yu et al., 2011). Several low-molecular chaperones (20 kDa chaperonin, 23.5 kDa HSP) were identified in our experiment. An increased relative abundance of several small HSPs including cytoplasmic as well as chloroplast and mitochondrial proteins was found in salt-treated tomato hypocotyls (Chen et al., 2009) and *Aster tripolium* leaves (Geissler et al., 2010). Increased relative abundance of STI1 protein, a stress-responsive phosphoprotein with two heat shock chaperonin-binding motifs and three TPR, was found in salt-treated rice panicles (Dooki et al., 2006) which points toward a regulatory network affected by salt stress since TPR-containing proteins were reported as being involved in myriads of processes including HSP90 signaling, gibberellin signaling and protein mitochondrial transport. GrpE protein homolog (M0YSD3, M0ZDK9) reveals an increase in salt-treated plants with respect to control plants in both *H. marinum* and *H. vulgare*. GrpE proteins are chaperones involved in ATP-dependent protein folding, interactions with client protein transit peptides and the client protein translocations to subcellular organelles such as mitochondrial matrix. Endoplasmin (M0VKI5, M0Z0U7) belongs to Hsp90 protein family and it is involved in ATP-dependent protein folding and translocation of client proteins into chloroplast stroma.

Protein disulfide isomerase (F2D284, M0UZB9) catalyzes a reversible oxidation of sulfhydryl groups in cysteine residues to form disulfide bridges thus affecting cellular redox homeostasis and protein conformation. PPI (M0WRW4, F2DA67 and M0WCQ8) catalyzes a reversible isomerisation between *cis*- and *trans*-conformation of peptide bonds next to proline residue. Enhanced PDI was found in drought-treated barley (Ashoub et al., 2013). A relatively decreased level of PDI (F2D284) was found in HVN with respect to HMN which corresponds with the findings of Mostek et al. (2015) who reported relatively higher PDI level in salt-tolerant barley cultivar with respect to the salt-sensitive one.

##### Defense response

Six LEA proteins (late embryogenesis abundant protein M0ZDL8; ABA inducible protein from LEA 4 family M0Z6A4; late embryogenesis abundant protein F2CRD9; late embryogenesis abundant protein D34 F2DNE8; late embryogenesis abundant protein 1 F2ECH4; dehydrin 8 M0UW32) and three PR proteins (pathogenesis-related protein M0Z8T3; germin F M0VST4; salt tolerant protein M0YJN2) were found only in salt-treated variants (HVN, HMN) while they were absent in control variants (HVC, HMC). An enhanced accumulation of dehydrin proteins in salt-treated *H. vulgare* was found in our previous study (Kosová et al., 2015) as well as in durum wheat (Brini et al., 2007). It is known that *H. vulgare* genome encodes 13 dehydrin genes out of which *Dhn1* to *Dhn11* are expressed in vegetative tissues (Choi et al., 1999) while *Dhn12* reveals an embryo-specific expression (Choi and Close, 2000) and *Dhn13* reveals an anther-specific expression (Rodriguez et al., 2005). Barley dehydrins belong to four structural types – K_n_ type (DHN5), SK_n_ type (DHN8), K_n_S type (DHN13) and Y_x_SK_n_ type (DHN1,2,3,4,6,7,9,10,11,12; Tommasini et al., 2008). Two major groups of dehydrin proteins, high-molecular dehydrins and low-molecular dehydrins, were detected in both salt-treated barley species on the immunoblots (**Figure 2A**). Of high-MW dehydrins, only one band corresponding to DHN5 protein was detected in *H. vulgare* while two high-molecular bands were detected in *H. marinum*. Of low-molecular dehydrins, four distinct bands were detected in *H. vulgare* while five were detected in *H. marinum*. Proteomic analysis revealed the presence of acidic SK_3_ dehydrin 8 in both barley species. It is known that both *Dhn5* and *Dhn8* genes are significantly induced by cold; however, they can also be induced by dehydration stresses and ABA (Choi et al., 1999; Tommasini et al., 2008; Kosová et al., 2013c, 2014b). The majority of *H. vulgare* dehydrins are of Y_x_SK_n_ type, relatively low-MW (14–30 kDa) and are induced by dehydration and ABA (Choi et al., 1999; Tommasini et al., 2008). A similar pattern of dehydrin expression seems to be found in halophytic *H. marinum*. Immunoblot results obtained on dehydrin proteins represent LC-MS/MS data validation since dehydrins as LEA proteins were detected only in salt-treated samples (HVN, HMN).

Germin F (M0VST4) was found in both salt-treated barley species (HVN, HMN) while it was absent in control variants (HVC, HMC). An increased abundance of germin-like proteins was reported in salt-treated leaves of barley (Fatehi et al., 2012), in barley response to powdery mildew (Zhou et al., 1998), in barley root exposed to salinity (Hurkman et al., 1994), aluminum (Tamás et al., 2004), as well as cadmium (Valentovicová et al., 2009), in cold-treated winter wheat crowns (Kosová et al., 2013b) and in salt-treated *Arabidopsis* roots (Jiang et al., 2007).

Universal stress protein (F2DHC4) is known to mitigate various abiotic stress impacts (Udawat et al., 2016); however, a precise role of plant USP proteins in stress protection remains largely unknown. Constitutive presence of USP protein in *H. marinum* while its absence in *H. vulgare* may contribute to superior salt tolerance of *H. marinum*.

##### Redox metabolism

Twenty-three proteins identified include enzymes involved in cleavage of superoxide anion radical such as cytosolic Cu/Zn superoxide dismutase (Cu/Zn-SOD M0VCA1, K4KCI3, M0YTZ0, M0VYA3), enzymes involved in cleavage of hydrogen peroxide (peroxidases POX M0X820, M0X558, F2CTB8, M0XHU1, M0YYV7; and peroxiredoxins Prx M0VDA7, M0X4Z3), enzymes involved in cleavage of lipid peroxides (lipoxygenases M0VUI3, M0Y2M0) as well as enzymes of ascorbate-glutathione cycle APX (M0UT11, M0X2A1, and M0YH83; monodehydroascorbate reductase MDAR F2D5M0). The majority of the proteins (17 out of 23) involved in redox metabolism were identified in salt-treated *H. marinum* (HMN) indicating an active stress acclimation. Fine regulation of ROS levels in plant cells represents an efficient tool in coordination of plant stress response (Suzuki et al., 2012).

Cystathione β-synthase domain-containing protein CBSX3 (M0Z0Z8) was found in all variants except for HVC. CBS domain proteins are involved in regulation of thioredoxin activity thus affecting cellular redox homeostasis (Bertoni, 2011). Previous study has reported improved salinity, redox and heavy metal tolerance in transgenic tobacco overexpressing rice CBS domain protein (Singh et al., 2012).

Lactoylglutathione lyase (F2CQP8) reveals glyoxalase activity using glutathione as a cofactor. It is known to be involved in glutathione-dependent detoxification of methylglyoxal which is a toxic byproduct of carbohydrate and amino acid metabolism. Lactoylglutathione lyase was detected only in HMC which corresponds to a constitutively enhanced level of this enzyme in roots of salt-tolerant barley cultivar Morex with respect to salt-sensitive barley cultivar Steptoe (Witzel et al., 2009) and indicates constitutively enhanced salt tolerance of *H. marinum* with respect to *H. vulgare*.

#### Regulatory Proteins

Cdc48 is cyclin involved in regulation of cell cycle and cell division. Cdc48 (M0Z3K0) was detected in HVC only which is consistent with a decline in Cdc48 observed in salt-treated halophyte *N. sphaerocarpa* (Chen et al., 2012).

Several glycine-rich RNA binding proteins (GRP; M0XEV2, F2DKA4, and M0WJV7) and low-temperature-responsive RNA binding protein (M0V3Q4) were identified, mostly in HMN. Small GRPs are known to be involved in the regulation of transcription and RNA processing. Several GRPs were reported to function as repressors of the major flowering repressor FLC and the members of an autonomous flowering pathway in *A. thaliana* (Quesada et al., 2005). Increased GRPs were found to be associated with vernalization fulfillment and a transition to flowering in winter wheat (Rinalducci et al., 2011) as well as in *A. thaliana* (Streitner et al., 2008). Increased abundances of several GRPs were reported in stress-treated plants such as in transgenic *A. thaliana*, poplar, and *Nicotiana tabacum* under salt and flooding stresses, respectively (Kwak et al., 2005; Durand et al., 2010; Wang et al., 2012).

Ricin B lectin (F2DIC8, M0VWY2) belongs to lectins which are glycoproteins involved in saccharide signaling as well as in regulation of cell development. Ricin B lectin was found to be increased in cold-treated winter barley and winter wheat crowns, respectively (Hlaváčková et al., 2013; Kosová et al., 2013b).

#### Apoptosis-Related Proteins

Proteins involved in regulation of PCD include seven proteins identified, three of them found specifically in HVN variant. The proteins specifically found in HVN include apoptotic chromatin condensation inducer in nucleus (F2E093) and FAS associated factor 2B (M0YTK2) which is involved in the interaction of FAS antigen with FAS ligand leading to initiation of apoptotic processes. The FAS associated factor binds to the FAS antigen thus enhancing its interaction with FAS ligand and inducing signaling processes leading to apoptosis. The presence of these proteins specifically in HVN indicates induction of processes leading to PCD in *H. vulgare* crown tissues by high salt stress. Plasminogen activator inhibitor 1 RNA-binding protein (M0WMM0, M0YZ72, and M0YVX6) is, according to GO annotation, involved in apoptotic processes (GO: 0042981) and regulation of mRNA stability (GO: 0043488). Guanine-nucleotide binding protein (G protein) subunit β (F2DSU6) is a phosphoprotein with WD-repeat motif which is known to be induced by exogenous ABA and is involved in a variety of cellular processes including signal transduction, transcription regulation, cell cycle regulation and apoptosis. Guanine-nucleotide binding protein subunit β was reported to be increased in salinity- and drought-treated rice (Dooki et al., 2006; Ke et al., 2009), respectively.

Translationally controlled tumor protein homolog (TCTP; F2DWT1) was identified in all experimental variants revealing a significantly increased level in HMN with respect to HVN. It is known that TCTP are calcium-binding proteins which are involved in an inhibition of p53 tumor suppressor-dependent apoptosis by binding to p53 thus down-regulating p53 activity. The obtained results thus indicate better suppression of apoptotic processes in HMN when compared to HVN. An increased abundance of TCTP was reported in barley plants exposed to salinity (Mostek et al., 2015) and drought (Ghabooli et al., 2013).

#### Structural Proteins

##### Cell adhesion

Out of eight proteins categorized to cell adhesion, four proteins (M0X5B5, M0WI23, M0XHE6, and M0YDA0) were identified as ankyrins (ankyrin-1 and ankyrin repeat domain-containing protein 2) which are known to be involved in anchoring integral membrane proteins in plasma membrane and assisting their attachment to spectrin-associated membrane skeleton. Other three proteins were identified as fasciclin-like proteins (fasciclin-like protein FLA5 M0Z9S4; fasciclin-like protein FLA10 M0W0B1; fasciclin-like protein FLA15 M0Z3D1). Fasciclins are secreted or membrane-anchored glycoproteins involved in cell wall architecture and biomechanics affecting plant stem strength (MacMillan et al., 2010). An increase in fasciclin-like protein FLA15 was found in HMN with respect to HMC while a relatively decreased level of ankyrin-repeat domain containing protein 2 was found in HVC with respect to HMC indicating both genotype-specific and salinity-induced alterations in cell biomechanical properties.

##### Cellular transport

Increased levels of Na^+^ lead to enhanced Na^+^ sequestration into vacuole. Vacuolar proton ATPase subunit E involved in Na^+^/H^+^ antiport was found in HMN. V-ATPases were reported to be increased upon salinity (Wang et al., 2008; Pang et al., 2010) and their activity is supposed to be regulated by SOS2 component of SOS1/SOS2/SOS3 signaling pathway (Batelli et al., 2007) and by glycolytic enzymes aldolase and ENO (Barkla et al., 2009).

Processes associated with mRNA and protein transport across nuclear pores are regulated by small GTPases from Ran family (Ran GTPases) and related proteins involved in Ran GTPase activation. Two Ran GTPase activating proteins (Ran GTPase-activating protein 1-like M0X1Z2; Ran-specific GTPase-activating protein 2 M0YLZ9) and one Ran-binding 1-c like protein (M0ZCE0) were identified in salt-treated variants (HVN, HMN) which is consistent with the finding that GTP-binding nuclear protein Ran1 was found increased in salt-treated shoots of *A. lagopoides* (Sobhanian et al., 2010).

Non-specific lipid transfer proteins are small proteins with a hydrophobic lipid binding site which are involved in lipid transfer from donor membranes such as endoplasmic reticulum to acceptor membranes such as chloroplasts, mitochondria, peroxisomes, and glyoxysomes. LTPs are also found in cell walls where they are involved in cuticle formation. LTPs were reported to be induced by several pathogens and environmental stresses including drought and salinity in wheat (Jang et al., 2004). Two nsLTPs (Q5UNP2, F2CY84), one lipid binding protein precursor (M0Z7W8) and one protein with sterol-binding domain (M0V4I6) were identified in salt-treated variants (HVN, HMN).

##### Cytoskeleton

Components of both microfilamental (actin cytoskeleton-regulatory complex protein PAN1 M0YG53; actin depoly merizing factor 4 F2DY31) and microtubular (tubulin alpha M0YMF1; β tubulin 6 A5CFY9; tubulin folding cofactor B F2CRA1) cytoskeleton were found in the samples; however, except for actin depolymerizing factor 4 found in salt-treated plants of both barley species (HVN, HMN), other cytoskeleton-associated proteins were reliably detected in one variant only. A decline in β tubulin and γ tubulin-interacting protein was found in salt-treated *N. sphaerocarpa* (Chen et al., 2012).

## Conclusion

**FIGURE 6:**
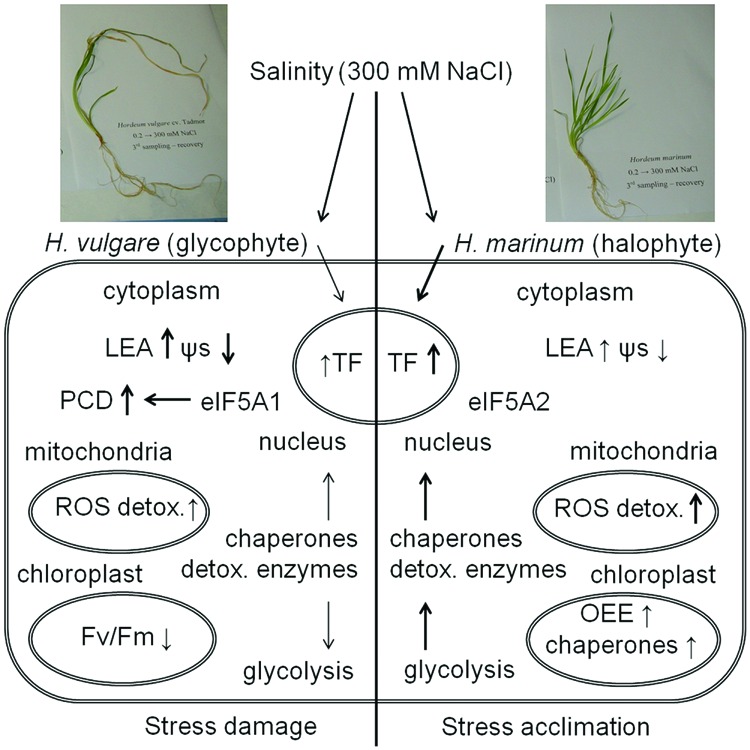
**A schematic summary of plant response to high salinity (300 mM NaCl) in crown tissues of *H. vulgare*, a glycophyte, and *H. marinum*, a halophyte**. ↑ means an increased protein relative abundance in salt-treated variant with respect to control while ↓ means a decreased protein relative abundance in salt-treated variant with respect to control. The intensity of the arrows indicates quantitative differences in physiological characteristics and protein levels between *H. vulgare* and *H. marinum*. See the abbreviations list and the text for abbreviation explanation.

The present study has revealed a differential response to high salinity of 300 mM NaCl between cultivated barley *H. vulgare* cv. Tadmor and a halophytic wild barley *H. marinum* (**Figure 6**). *H. marinum* revealed constitutively enhanced tissue dehydration with respect to *H. vulgare*. In contrast, *H. vulgare* revealed lower osmotic potential and higher levels of osmolytes such as proline and dehydrins with respect to *H. marinum* which is consistent with the results of previous studies and corresponds with enhanced Na^+^ vacuolar accumulation in salt-treated *H. vulgare* shoot tissues as reported previously (Garthwaite et al., 2005; Islam et al., 2007).

Proteomic analysis revealed significant differences between proteomes of control and salt-treated plants as well as between both barley species. Proteins identified specifically in control plants (HVC, HMC) include proteins associated with active biosynthetic processes including cytoplasmic and organellar protein biosynthesis (40S ribosomal protein SA, 60S acidic ribosomal protein P0, 60S ribosomal protein L22-2; translation elongation factor Tu); proteins involved in carbohydrate biosynthesis (sucrose synthase), fatty acid biosynthesis [enoyl-(acyl-carrier-protein) reductase (NADH)], chlorophyll biosynthesis (magnesium chelatase 40 kDa subunit) as well as proteins involved in amino acid biosynthesis (cysteine synthase, serine hydroxymethyltransferase) and amino acid-oxoacid interconversions (aspartate aminotransferase). In contrast, proteins identified specifically in salt-treated plants (HVN, HMN) include proteins involved in ATP biosynthesis and metabolism (mitochondrial ATP synthase precursor, soluble inorganic pyrophosphatase 1) indicating enhanced need for immediately available energy in the form of ATP during the process of an active stress acclimation. Moreover, several proteins involved in stress and defense responses and redox metabolism (germin F, LEA protein D34, dehydrin 8, peroxidase 2) were found only in salt-treated samples.

*Hordeum vulgare* subjected to salinity reveals severe damage under high-salinity stress (300 mM NaCl) indicated by a presence of several proteins involved in apoptotic processes (apoptotic chromatin condensation inducer in the nucleus; FAS-associated factor 2-B) and proteins involved in protein ubiquitination resulting in protein targeting to proteasomal degradation (E3 UMF1-protein ligase 1 homolog; deubiquitination-protection protein dph1). A relative decrease in several proteins associated with energy metabolism such as glycolytic proteins ALDO, ENO, PGM, TPI indicates serious damage of HVN metabolism under high salinity. Changes at proteome level thus indicate processes leading to tissue damage and PCD. Our previous study indicates that *H. vulgare* cv. Tadmor plants subjected to one-step transfer to high salinity still not fully recovered after 1 week of recovery treatment when compared to Tadmor plants subjected to a gradual salt acclimation (Kosová et al., 2015). In the present study, the data obtained by proteomic analysis indicate that an one-step transfer to 300 mM NaCl will finally result in irreversible crown tissue damage in *H. vulgare* cv. Tadmor.

In contrast, *H. marinum* transferred to 300 mM NaCl reveals an active acclimation to high salinity indicated by enhanced levels of several proteins involved in energy metabolism (ATP metabolism), protection of intracellular structures (proteins involved in protein folding, redox reactions), and even proteins involved in sensitive processes of energy metabolism such as photosynthesis (OEE proteins in PSII). These proteins indicate an enhanced protection of stress-sensitive processes such as photosynthesis in halophytic *H. marinum* with respect to glycophytic *H. vulgare*. Enhanced levels of proteins involved in cleavage of macroergic phosphate bonds (adenylate kinase A, soluble inorganic pyrophosphatase) indicate enhanced need for immediately available energy during salt stress acclimation. The presence of several isoforms of ROS scavenging enzymes as well as detoxification enzymes such as CBS domain-containing protein CBSX3, USP, cyanate hydratase, and lactoylglutathione lyase indicates fine tuning and efficient decomposition of toxic byproducts of cellular metabolism. Moreover, enhanced levels of dehydration stress-responsive TFs such as NAC and bZIP TFs underlie enhanced levels of several stress-responsive proteins indicating an active stress acclimation. Differential states of *H. vulgare* and *H. marinum* subjected to high salt levels are also indicated by the presence of different isoforms of eIF-5A with eIF-5A1 isoform involved in apoptosis induction in *H. vulgare* while eIF-5A2 isoform involved in cell division induction in *H. marinum*.

## Author Contributions

LM performed protein identification using nanoLC-ESI-Q-TOF and analysed the data. RH analyzed protein MS/MS data. KK designed the whole experiment, determined plant physiological characteristics, prepared literature review and wrote the manuscript. PV and IP participated on the manuscript and figure captions preparation.

## Conflict of Interest Statement

The authors declare that the research was conducted in the absence of any commercial or financial relationships that could be construed as a potential conflict of interest.

## Footnotes

1 http://pfam.xfam.org

2 http://www.geneontology.org/

## ABBREVIATIONS

ALDO: fructose-bisphosphate aldolase
APX: ascorbate peroxidase
bZIP: basic leucine zipper (protein motif)
CBS: cystathione β synthase
cdc48: cell division cycle 48 (protein)
DHN: dehydrin
eEF: eukaryotic elongation translation factor
eIF: eukaryotic initiation translation factor
ENO: enolase
ENR: enoyl(acyl-carrier-protein) reductase (NADH)
ESI: electrospray ionization
*F*_v_/*F*_m_: variable-to-maximum chlorophyll fluorescence
GAP: GTPase activating factor
GAPDH: glyceraldehyde 3-phosphate dehydrogenase
GEF: GDP/GTP exchange factor
GRP: glycine-rich protein
HMC: *Hordeum marinum, control plants*
HMN: *H. marinum, salt-treated plants*
HSP: heat-shock protein
HVC: *Hordeum vulgare, control plants*
HVN: *H. vulgare, salt-treated plants*
LC-MS: liquid chromatography mass spectrometry
LEA: late embryogenesis-abundant (protein)
NAC: nascent polypeptide associated complex
nsLTP: non-specific lipid transfer protein
OEC: oxygen evolving complex
OEE: protein component of oxygen evolving complex
PCD: programmed cell death
PDI: protein disulfide isomerase
PGK: 2,3-bisphosphoglycerate kinase
PGM: 2,3-bisphosphoglycerate-independent phosphoglycerate mutase
POX: peroxidase
PP2C: protein phosphatase 2C
PPI: peptidyl-prolyl *cis-trans* isomerase
PR: pathogenesis-related (protein)
Prx: peroxiredoxin
PTM: posttranslational modification
PWC: plant water content
RC PSII: reaction centre of photosystem II
ROS: reactive oxygen species
SAM: S-adenosylmethionine
SOD: superoxide dismutase
STI: stress-induced (protein)
TCA: tricarboxylic acid (cycle)
TCTP: translationally-controlled tumour protein
TF: transcription factor
TPI: triose phosphate isomerase
TPR: tetratricopeptide repeat (protein motif)
USP: universal stress protein
*W*_D_: tissue dry weight
*W*_F_: tissue fresh weight
*W*_T_: tissue turgescent weight
WSD: water saturation deficit
ZFHD: zinc-finger homeodomain (protein motif)
ψ_s_: osmotic potential
